# Cutaneous mucormycosis in systemic lupus erythematosus: a case report

**DOI:** 10.3389/fimmu.2025.1602639

**Published:** 2025-09-10

**Authors:** Shiyang Li, Ting Meng, Yuqiu Lin, Dong Xie, Zhihua Li, Qing Jiang

**Affiliations:** ^1^ Dermatology Hospital of Jiangxi Province, Nanchang, China; ^2^ Jiangxi Provincial Clinical Research Center For Skin Diseases, Nanchang, China; ^3^ Candidate Branch of National Clinical Research Center for Skin Diseases, Nanchang, China; ^4^ Dermatology Institute of Jiangxi Province, Nanchang, China; ^5^ The Affiliated Dermatology Hospital of Nanchang University, Nanchang, China; ^6^ School of Public Health, Nanchang University, Nanchang, Jiangxi, China; ^7^ Department of Medical Cosmetology, The Second Affiliated Hospital of Nanchang University, Nanchang, Jiangxi, China

**Keywords:** systemic lupus erythematosus, *Rhizopus arrhizus*, mucormycosis, skin, cutaneous mucormycosis

## Abstract

Mucormycosis is a life-threatening angioinvasive fungal infection caused by the Mucorales acidophilic fungi. In the article we report a case of cutaneous mucosporidiosis caused by infection with *Rhizopus arrhizus* in a patient with systemic lupus erythematosus (SLE). We analyze the causes of infection and death and concluded that cutaneous mucormycosis is a severe and refractory infection that has higher mortality in immunocompromised individuals or those with immune dysfunction. Early diagnosis and proactive treatment are paramount for a favorable prognosis.

## Introduction

Mucormycosis is an angioinvasive fungal disease that is characterized by tissue infarction and necrosis (1, 2). *Rhizopus* is the most common pathogen of mucormycosis, accounting for approximately 70% of all cases. The major risk factors include neutropenia, diabetic hyperglycemia, diabetic ketoacidosis, hypoimmunity, etc. (3) with the respiratory tract or skin being the most common infection pathway (4). Cutaneous mucormycosis is more prevalent in the nosocomial and pulmonary types, but cutaneous mucormycosis is also increasing year by year. Ren et al. (5)reported a case of herpes zoster secondary to cutaneous mucormycosis in a patient with diabetic ketoacidosis. In addition, cutaneous mucormycosis can also be seen in immunocompetent patients. Shingde et al. (6) reported a case of a patient who was immunocompetent and had no predisposing risk factors, and who also developed cutaneous mucormycosis with only recently minor skin trauma.

Systemic lupus erythematosus (SLE) is a systemic autoimmune disease characterized by systemic multisystemic and multiorgan involvement, recurrent relapses and remissions, and the presence of large amounts of autoantibodies in the body (7). It results in a state of severe immunosuppression due to T-lymphocyte dysfunction, neutropenia, lymphopenia, and alterations in complement pathways (8), which increases the risk of opportunistic infections. Currently, infections caused by mucormycosis in patients with SLE are mainly manifested as rhinocerebral type, renal type, gastrointestinal type, and disseminated type. However, there are relatively few reports on cutaneous type mucormycosis. The reported pathogens are *Absidia corymbifera* (9), *Rhizopus microsporus* (10), *Rhizopus azygosporus* (11), etc., while *Rhizopus arrhizus* infections of SLE patients have not been found or not clearly indicated. This study reports cutaneous mucormycosis caused by *Rhizopus arrhizus* infection in a male patient with SLE.

## Case presentation

**Figure 1:**
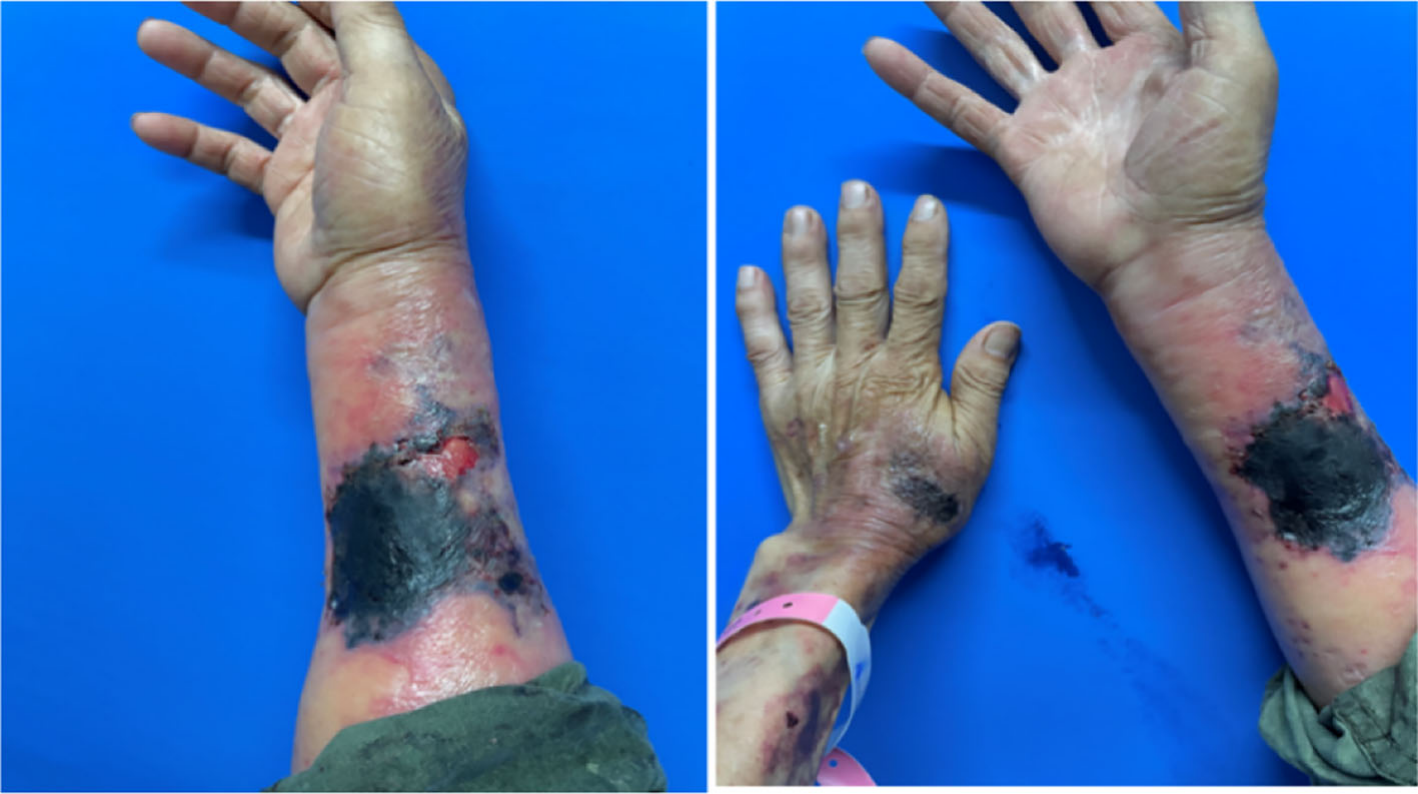
Egg and palm-sized patchy necrosis observed on the back of the left hand and the right forearm, covered with a thick black scab, surrounded by diffuse edematous erythema with ill-defined boundaries. A part of the scab on the right forearm shed, revealing a red basal ulcer surface. A little exudation and scabbing were observed.

A 68-year-old male farmer presented with a 20-day history of ulcers, necrosis, redness, swelling, and pain on both forearms. Erythema on the right forearm, which developed 20 days after sustaining an injury during farm work in the fields, but no special treatment was performed. It rapidly expanded with ulceration and necrosis in the center covered by a thick black scab, accompanied by slight swelling and pain. He was treated with dexamethasone and cefuroxime axetil for anti-inflammatory and anti-infective treatments at a local hospital; however, the effects were unsatisfactory. Then, 6 days later, diffuse edematous erythema developed on the left forearm. His past medical history was significant for chronic renal insufficiency for 10 years(long-term use of “Shenshuaining tablets”), arthritis for 3 years(received intermittent use of “triamcinolone tablets”), and thrombocytopenia for 4 years(medication history is unknown.). Patients often have mouth ulcers. His physical examination findings were as follows: temperature was 36.7°C, pulse was 86 beats/min, respiration rate was 20 breaths/min, and blood pressure was 127/64 mmHg; he had regular heart rate and no murmur; the heart boundary was enlarged to lower left; and pulmonary breath sounds were coarse bilaterally without rales. The dermatological examination revealed the following findings: Egg and palm-sized patchy necrosis was noted on the back of the left hand and right forearm; it was covered with a thick black scab and surrounded by diffuse edematous erythema with ill-defined boundaries. A part of the scab on the right forearm shed and a red basal ulcer surface was evident. A little exudation and scab could be seen (**Figure 1**). On admission, platelet counts(PLT) were markedly decreased and the levels of C-reactive protein and urinary protein have significantly increased (**Table 1**). The coagulation function test results was unremarkable. Autoantibody assay revealed that antinuclear antibody (type SH 1:1000), anti-double-stranded DNA antibody, anti-SSA antibody, and antiphospholipid antibody tested positive. Abdominal color Doppler ultrasonography revealed splenomegaly and prostatic hyperplasia. Furthermore, thoracoabdominal computed tomography angiography (CTA) showed mild atherosclerosis of the thoracoabdominal aorta, small aortic arch ulcer, local emphysema, scattered chronic inflammatory lesions in both lungs, and partial small intestinal wall thickening. Craniocerebral magnetic resonance angiography demonstrated a few ischemic lesions in the brain, senile brain changes, and a left occipital subarachnoid cyst.

**Table 1:** Laboratory findings of the patient on the day of admission.

Laboratory index	Result	Reference range
WBC (×10^9^/L)	11.47	4~10
RBC (×10^9^/L)	2.90	3.5~5.5
PLT (×10^9^/L)	52.00	100~300
LYC (×10^12^/L)	0.32	0.8~4
NE%	90.30	50~70
Hb (g/dL)	99.00	110~160
CRP(mg/L)	68.10	0.1~8.2
ESR(mm/h)	45.00	0~20
Urine protein	2+	–
24-hour urine protein(mg)	2047.98	<150
Albumin (g/L)	29.10	33~55
Serum Creatinine(μmol/L)	152.00	57~97
C3(g/L)	0.63	0.8~1.5
C4(g/L)	0.11	0.2~0.6
Alpha-Fetoprotein(ng/mL)	3.00	<7.00
Carcinoembryonic Antigen(ng/mL)	4.92	<4.50
Carbohydrate Antigen 19-9(μ/mL)	<1	<30.00
Carbohydrate Antigen 125(μ/mL)	11.26	<35.00
Total Prostate-Specific Antigen(ng/mL)	1.64	<4.00
Squamous Cell Carcinoma Antigen(ng/mL)	0.53	0~1.80

WBC, white blood count; RBC, red blood cell; PLT, platelet count; LYC, lymphocyte count; NE%, neutrophil percentage; Hb, hemoglobins; CRP, c-reactive protein; ESR, erythrocyte sedimentation rate; C3, component 3; C4, component 3; -, negative.

**Figure 2:**
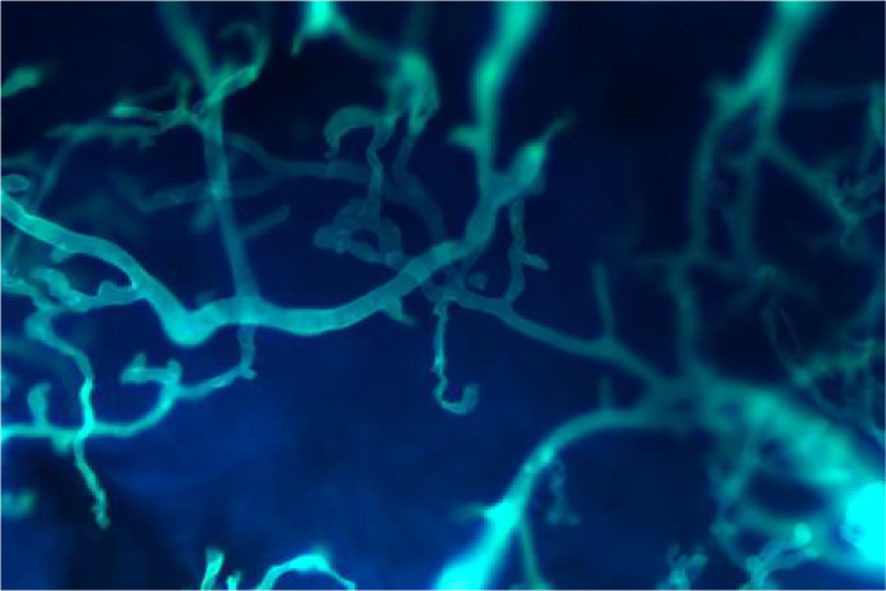
Fungal microscopy revealed wide and transparent hyphae with no septum were seen, and the hyphae were irregularly branched, twisted, and folded into ribbons (Calcofluor white stain ×400).

**Figure 3:**
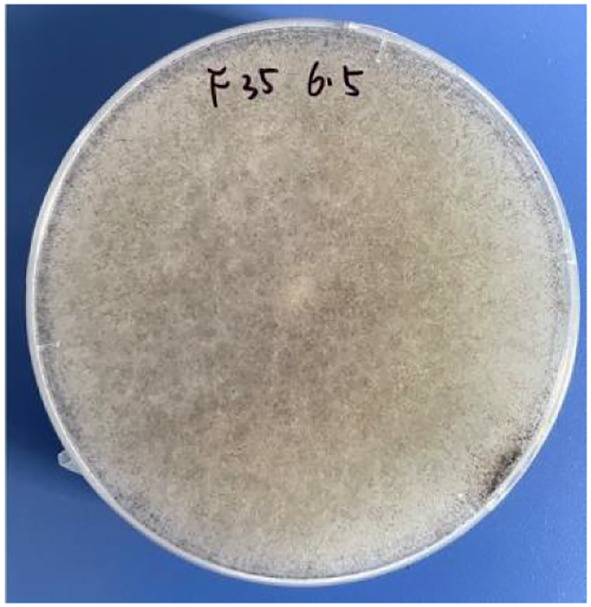
Fungal culture showed that the front of the colony initially appeared as white cotton wool; then, gradually scattered small black spots were observed in aerial hyphae. Finally, the colony turned gray-brown.

**Figure 4:**
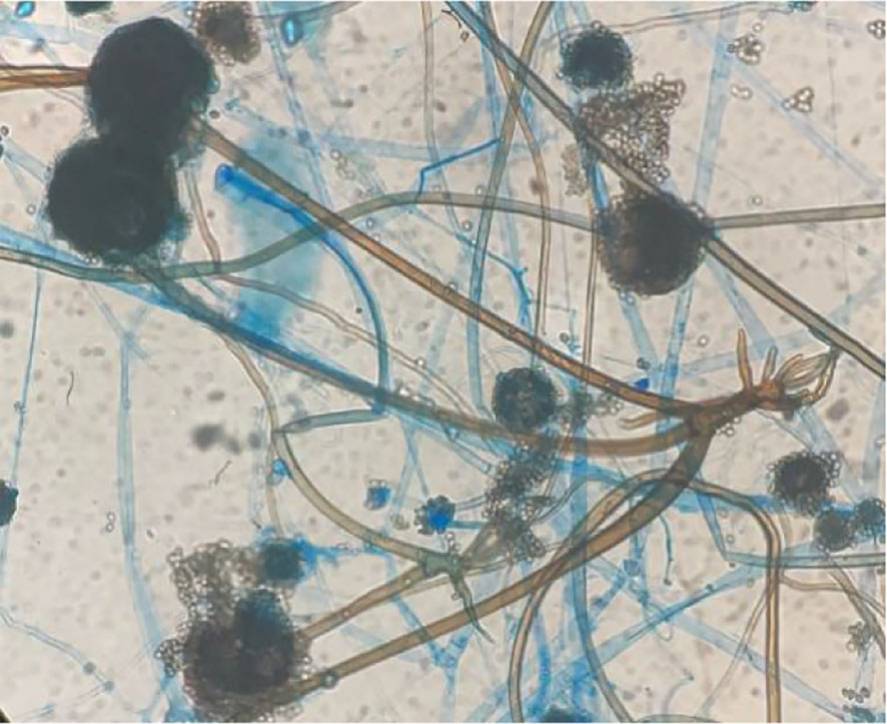
Potato dextrose agar under a small culture microscope revealed wide brown hyphae; sporangia, sporangiophores, and rhizoids were opposite, and the rhizoids were underdeveloped (Cotton blue stain ×400).

**Figure 5:**
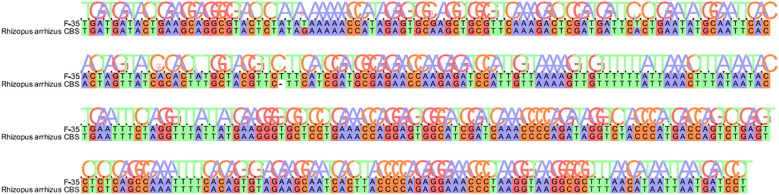
Results of sequence comparison with NCBI strains.

Fungal microscopy of skin lesions conducted three times revealed wide and transparent hyphae with no septum. The hyphae displayed irregular branching, twisting, and folding into ribbons (**Figure 2**). Subcutaneous secretions of multiple sites were submitted for fungal culture on sabouraud dextrose agar at 28°C for 4–7 days and displayed rapid colony growth. The front of the colony initially resembled white cotton wool, gradually revealing scattered small black spots in aerial hyphae, culminating in a gray-brown colony (**Figure 3**). Potato dextrose agar under a small culture microscope demonstrated wide brown hyphae, opposite sporangia, sporangiophores and rhizoids, and underdeveloped rhizoids. Morphologically, it was identified as *Rhizopus* (**Figure 4**). After extracting DNA from cultured strain, polymerase chain reaction amplification (PCR) was performed. The PCR reaction mixture (50 μL total volume) contained:2× Rapid Taq Master Mix (25μL), forward and reverse primers(each 1.5 μL; see **Table 2**), DNA template (4 μL), and ddH_2_O (18μL). The PCR cycling program was as follows:initial denaturation at 95°C for 5 min; followed by 30 cycles of denaturation at 95°C for 30 sec, annealing at 60°C for 30 sec, and extension at 72°C for 1 min; with a final extension at 72°C for 5 min. The resultant product was submitted to the Sangon Biotech (Shanghai) Co., Ltd. for sequencing. The sequencing results were uploaded to the National Center for Biotechnology Information (NCBI) database for blast search, and found the highest homology with the strain NR_103595.1. Applying Multiple sequence alignment (MSA) method to compare the regional sequence homology was 98.06% (**Figure 5**), combined with the results of fungal microscopic examination and fungal culture, it was determined that as *Rhizopus arrhizus*.

**Table 2:** PCR amplification primers.

Gene Name	primer sequence(5’-3’)	Primer length (bp)
ITS	ITS1:TCCGTAGGTGAACCTGCGG	19
	ITS4:TCCTCCGCTTATTGATATGC	20

Based on laboratory tests and clinical manifestations, we diagnosed cutaneous mucormycosis combined with systemic lupus erythematosus(SLE) and lupus nephritis. The patient received human immunoglobulin and fluconazole for antifungal therapy. Following the tissue culture confirmation of Rhizopus, the treatment plan was adjusted to include amphotericin B combined with posaconazole for antifungal therapy, along with muscular debridement, vacuum sealing drainage, and pedicled skin flap transplantation. Continuous monitoring of hepatorenal function, electrolytes, and blood sugar was implemented. Due to poor postoperative recovery, the right forearm developed muscle necrosis, cyanosis, poor arterial pulsation, and no obvious finger pressure response. Consequently, the patient underwent right upper extremity amputation under general anesthesia and was transferred to the intensive care unit for further care. Despite efforts, the patient and his family members opted to return to the local hospital for continuous treatment due to financial constraints, leading to the unfortunate demise of the patient during follow-up.

## Literature review

A retrospective analysis was performed on published case reports of mucormycosis infections in SLE patients by searching PubMed, EMbase, and Web of Science databases using the keywords “systemic lupus erythematosus, mucormycosis” from each database’s inception until July 2025, excluding cases with incomplete documentation or insufficient clinical data. The analysis identified 16 reported cases of mucormycosis in SLE patients in English literature. **Table 3** comprehensively presents the patients’ clinical characteristics, initial clinical manifestation, treatment, and outcome, showing 11 females (68.75%) and 5 males (31.25%) with a mean age at infection of 35.5 ± 14.75 years. There were 8 cases of nasoencephalic, gastrointestinal and renal forms with a mortality rate of 62.50%, 3 cases of disseminated forms with a mortality rate of 100.00%, and 5 cases of dermatologic forms with a mortality rate of 20.00%. Treatment outcomes revealed a 20.00% cure rate for amphotericin monotherapy and 66.67% for amphotericin combined with surgery or adjunctive medications.

**Table 3:** The clinical characteristics of patients with systemic lupus erythematosus complicated with mucormycosis as reported in the literature.

Authors	Year	Gender	Age(y)	Initial clinical manifestation	Mucormycosis clinical form	Diagnosis	Pathogen type	Treatment	Outcome
Saboya (12)	2022	Male	53	Subcutaneous nodules and joint pain	Vertebral	Biopsy	Rhizopus	Amphotericin + surgery	Recovered
Roche (9)	2021	Male	28	Skin ulcer	Cutaneous	Biopsy + tissue culture	Absidia corymbifera	Itraconazole	Recovered
Sigera (13)	2018	Female	29	Skin ulcer	Cutaneous	Biopsy + tissue culture	Saksenaea vasiformis	Amphotericin + surgery	Recovered
Kumar (14)	2013	Female	28	Pale complexion, swollen face and lower limbs	Rhinocerebral	Biopsy	Mucorales	Amphotericin + surgery	Recovered
Shenoi (10)	2009	Male	14	Fever, fatigue, swollen lymph nodes, and anemia	Gastrointestinal	Biopsy	Rhizopus microsporus	Amphotericin + Micafungin	Recovered
Salinas (15)	2010	Female	75	Posterior ocular optic neuritis	Rhinocerebral	Biopsy	Mucorales	Amphotericin	Died
Yu (16)	2006	Male	34	Difficulty in urination and painful urination	Renal	Biopsy	Rhizopus oryzae	Amphotericin	Died
Fujimoto (11)	2005	Female	54	Skin ulcer	Cutaneous	Biopsy	Rhizopus azygosporus	Amphotericin	Died
Mok (17)	2003	Female	36	Fever and joint pain	Rhinocerebral	Throat swab	Rhizopus	Amphotericin	Died
Alsuwaida (18)	2002	Female	39	Skin ulcer	Cutaneous	Biopsy	Rhizopus	Amphotericin	Recovered
Liu (19)	2000	Female	17	High fever, breathing difficulty, and lung abscess	Disseminated	post-mortem.	Zygomycota	Fluconazole	Died
Hosseini (20)	1998	Female	42	High fever, chills, and generalized body aches	Gastrointestinal	Biopsy	Mucorales	Amphotericin + surgery	Died
Dickinson (21)	1998	Female	36	Fever, cough, and difficulty breathing	Cutaneous	Biopsy + tissue culture	Rhizopus pu s rhi:::apudiformis.	Amphotericin + surgery	Recovered
Bloxham (22)	1990	Female	29	Abdominal pain and vomiting	Disseminated	post-mortem.	Rhizopus	None	Died
Escobar (23)	1990	Female	33	Fever, headache, and drowsiness	Cerebral	post-mortem.	Mucorales	None	Died
Wong (24)	1986	Female	21	Coughing accompanied by green phlegm	Disseminated	post-mortem.	Mucorales	None	Died
Our case	2025	Male	68	Skin ulcer	Cutaneous	Tissue culture + sequencing	Rhizopus arrhizus	Amphotericin + surgery	Died

## Discussion

Mucormycosis is an opportunistic infection caused by fungi of the class Zygomycetes, which may rapidly lead to death if timely diagnostic intervention is not made (25). The first case was documented in 1885 by Paltauf (26). Since then, its incidence has been on the rise, ranking as the third most common invasive fungal disease in immunosuppressed individuals, following candidiasis and aspergillosis. The global incidences of mucormycosis (per million people) in 2019 varied, with 95 cases in Portugal, 3 in the United States, 1.2 in Canada, 0.6 in Australia, and 140 in India (27). The infection is characterized by extensive vascular invasion, leading to thrombosis, tissue infarction, and necrosis. Clinically and anatomically, mucormycosis can manifest as rhinocerebral, pulmonary, cutaneous, gastrointestinal, disseminated, and other types (28), with rhinocerebral mucormycosis being the most prevalent (29). Cutaneous mucormycosis, constituting only 10% of cases, includes superficial, nodular, and gangrenous forms. Typically, it presents as a single, painful erythematous area, with severe cases progressing to life-threatening tissue necrosis, resulting in a 10% mortality rate; however, disseminated infection carries a staggering 94% mortality (30). Cutaneous mucormycosis is often associated with traumatic soil inoculation, burns, and intravenous infusion, breaching the skin barrier and leading to infections (31, 32). Our study reports cutaneous mucormycosis caused by Rhizopus arrhizus infection in an elderly male patient with SLE.

Our patient, who presented with a history of thrombocytopenia for 4 years, renal insufficiency for 10 years, and arthritis for 3 years, was admitted to the hospital with complaints of ulcers, necrosis, redness, swelling, and pain on both forearms for 20 days. Previously reported cases were mostly young-aged (Mean age 35.5 ± 14.75 years) women, which is slightly different from the present case. Relevant laboratory tests after admission revealed antinuclear antibody (type SH 1:1000), anti-double-stranded DNA antibody, anti-SSA antibody, and antiphospholipid antibody tested positive. According to the European League Against Rheumatism/American College of Rheumatology(EULAR/ACR) SLE classification 2019 criteria (33), SLE with lupus nephritis was diagnosed, and the SLE disease activity index (SLEDAI) score was 17 points. Therefore, it was speculated that trauma was the trigger for Rhizopus arrhizus infection in our patient, and SLE was the contributing factor of cutaneous mucormycosis. SLE is an autoimmune disorder of unknown cause, which is characterized by the production of multiple autoantibodies and multi-system target organ damage; it is more likely to occur in young women (34). Patients with SLE have immune function deficiencies, including chemotaxis, membrane recognition and microbial attachment, phagocytosis, oxidative metabolism, and interleukin-8 production by polymorphonuclear leukocytes (35). Furthermore, due to disease activity and the use of glucocorticoids and immunosuppressants, infection easily occurs, and the possibility of hospitalization due to opportunistic infections is 24 times that of the general population (36). When the SLEDAI score is >12 points (the SLEDAI score in our patient was 17 points), the risk of infection also increases (37). Among fungal infections in patients with SLE, the most common fungi are *Candida, Cryptococcus, Aspergillus, and Pneumocystis carinii* (34, 38), whereas *Rhizopus arrhizus* infection in patients with SLE is relatively rare.

Based on the clinical manifestations, laboratory tests, fungal microscopy, and tissue culture results of this patient, the diagnosis of SLE complicated with fungal infection was promptly established. Timely antifungal therapy, specifically amphotericin B combined with posaconazole, was initiated upon considering cutaneous mucormycosis caused by Mucorales based on multiple culture results. Despite aggressive treatment, including surgical debridement and other interventions, the prognosis remained poor. Several factors may have contributed to this outcome: 1) Delayed medical intervention. The extended 20-day duration before seeking medical treatment played a crucial role in the unfavorable prognosis. Delayed initiation of targeted treatment, coupled with hormone therapy prescribed by the local hospital during this period, likely exacerbated disease progression, highlighting the importance of early intervention in cases of cutaneous mucormycosis. 2) Combined with SLE. The confirmed diagnosis of SLE during this visit, coupled with the patient’s past medical history of renal insufficiency, thrombocytopenia, and arthritis, suggests the presence of SLE for several years. The active stage of SLE, evident from a SLEDAI score of 17 points, might have further compromised the patient’s immune function, contributing to an increased risk of infection. Furthermore, patients with active SLE have reduced T lymphocyte count, impaired phagocytic capacity, the presence of anti-Fc γ receptor autoantibodies and compromised T lymphocyte activities for various antigens, toxoids, and allogeneic antigens. Additionally, abnormalities in complement levels particularly reduced non-hereditary complements like C4 and reduced complement receptor types 1 and 2 (CR1 and CR2) further increase the vulnerability to infections, weakening the body’s autoimmune response and the ability to kill pathogenic microorganisms (35).

According to our statistics of previous cases, the mortality rate of cutaneous mucormycosis is not high, only 20%, and the mortality rate of disseminated type is 100%, but our patient still unfortunately passed away. By comparing with SLE combined with mucormycosis as reported by other scholars. In this case, the time from diagnosis to targeted treatment was at least greater than 4 weeks and the rash appeared first on the right hand and then spread to the left hand, making a disseminated infection suspicious, similar to that reported by Mok et al. (17) In addition, in terms of treatment modalities, we used fluconazole for antifungal therapy at the initial stage of treatment because the results of the pathogen were not yet available, and Fujimoto et al. (11) also failed to prioritize the use of amphotericin for treatment because of the nephrotoxic effects of amphotericin, these patients eventually died. However, in the cases reported by Saboya, Sigera, and Dickinson et al (12, 13, 21), they were both localized infections without distant dissemination and were treated with amphotericin in a timely manner, so both patients were fortunate to survive. Therefore, if a suspected case is found during clinical diagnosis and treatment, the pathogenetic examination should be completed as soon as possible to make a clear diagnosis and active treatment and adequate course of treatment, which can significantly improve the prognosis of the patient; if it progresses to disseminated infection, the prognosis is extremely poor, and the mortality rate is significantly higher.

Currently, the preferred medications for cutaneous mucormycosis are amphotericin B and liposomal amphotericin B (29), with the first-line treatment regimen being liposomal amphotericin B combined with extensive surgical debridement (39). According to our statistical data, the combined use of amphotericin B and surgical debridement has an effective rate of up to 66.67%. New-generation triazole antifungals such as posaconazole and isavuconazole have shown effectiveness against Mucorales infection (40). While calcineurin inhibitors are more likely to enhance the activity of isavuconazole against Mucorales fungi than inhibitors of the mTOR pathway, and further investigation is warranted based on *in vitro* experimental results (41). Despite advancements in antifungal therapy, mucormycosis remains a severe and refractory infection with a lower overall survival rate. Early diagnosis and active treatment remain pivotal in improving the prognosis of individuals afflicted by this challenging condition.

In conclusion, cutaneous mucormycosis is a severe and refractory infection that has higher mortality in immunocompromised individuals or those with immune dysfunction. Early diagnosis and proactive treatment are paramount for a favorable prognosis.

## Data Availability

The raw data supporting the conclusions of this article will be made available by the authors, without undue reservation.
